# Optimizing the Pore Structure of Bio-Based ACFs through a Simple KOH–Steam Reactivation

**DOI:** 10.3390/ma9060432

**Published:** 2016-05-31

**Authors:** Yuxiang Huang, Erni Ma, Guangjie Zhao

**Affiliations:** College of Materials Science and Technology, Beijing Forestry University, Beijing 100083, China; yxhuang@utexas.edu (Y.H.); maerni@bjfu.edu.cn (E.M.)

**Keywords:** biomass materials, activation carbon fiber, reactivation, microporosity, iodine adsorption

## Abstract

Highly microporous bio-based activated carbon fibers (ACFs) were prepared through a simple reactivation method. Sawdust, as the starting material, was liquefied and melt-spun to produce the precursor fibers. Then, the precursor fibers were activated by KOH and reactivated by steam. By varying the conditions of the two activation processes, the formation mechanism of the pore structure was studied, and the result showed that steam reactivation has a positive effect on the development of microporosity. The sample with the optimal condition exhibited the highest specific surface area of 2578 m^2^·g^−1^ as well as the largest pore volume of 1.425 cm^3^·g^−1^, where micropores contributed 70.3%. Due to its excellent texture properties, the ACF exhibited a high adsorption capacity of 1934 mg/g for iodine.

## 1. Introduction

Adsorption is an efficient method for removing a wide range of pollutants from water and air for recovery and purification because of its low cost and simple operation [1]. As a type of carbon nanoporous absorbent, activated carbon fiber (ACF) has attracted considerable interest on account of its excellent properties as compared with conventionally activated carbon, such as good flexibilities in fabricating cloth, felt, paper and other textile forms, high adsorption capacity and rates due to the high specific surface area, and narrow pore size distributions [2,3].

As is well known to us, pore texture and surface chemistry are the two essential factors affecting the adsorption capability of ACF, both of which are determined by the fabrication process as well as the precursor selected [4,5]. Thus, the raw material plays an important role in producing high-performance ACF.

Sawdust, as one of the lignocellulosic biomasses, is a by-product from the mechanical milling or processing of wood into various usable sizes [6]. This lignocellulosic material is a promising precursor for ACF production. The earliest attempt to utilize wood sawdust as a precursor for carbon fiber began in 2010 [7]. With this attempt and the work thereafter, a method for the preparation of sawdust-based ACF was developed using melt spinning sawdust-based fiber as the precursor. Microporous sawdust-based ACFs with a high specific surface area (SSA) were successfully prepared via steam or KOH activation [8,9].

With the extension of ACF’s applicability, such as in macromolecule adsorption [10] and energy storage [11], recent requirements for ACFs have become greater, and ACFs with uniform pore structure cannot meet various demands. Our previous study found that, although ACFs prepared from single steam activation had a high SSA above 2000 m^2^·g^−1^, their yield was very low, and their pore size distribution (PSD) was narrow [8]. By comparison, ACFs prepared from single KOH activation had higher yield and wider PSD [12]. Moreover, mesoporous ACFs can be obtained by applying very low KOH/fiber ratio (1:1) during the single KOH activation [12]. Thus, in ensuring high SSA and yield, combining steam activation with KOH activation to prepare ACFs with well-controlled porosity is expected. There are two options for the reactivation method by adjusting the order. One is a first steam activation, followed by a second KOH activation (steam–KOH reactivation). The other is a reverse way (KOH–steam reactivation). It was reported that reactivation is a simple and effective way to further develop the ACF porosity in many studies. Miyamoto *et al.* used three activating agents (Na_2_HPO_4_, Ca(NO_3_)_2_, and K_2_CO_3_) to reactivate pitch-based ACFs and found all the activating agents were able to add the mesoporosity except K_2_CO_3_ [13]. The adsorption rate of the resultant mesoporous ACFs by Ca(NO_3_)_2_ reactivation to methylene blue was increased by more than 10 times [14]. Wang *et al.* conducted steam reactivation of polyacrylonitrile-based ACFs and studied the changes in SSA and surface chemistry of the ACFs [15]. However, their focus was mainly on the reactivation process using the ACFs as starting materials rather than the order of the two activation processes using precursor fibers as starting materials. Our previous study revealed that ACFs prepared by reactivation combining steam and KOH activation with different orders exhibited different pore structures [16]. In other words, reactivation with different orders had different pore-forming mechanisms. Recently, we published a study on the preparation of ACFs by steam–KOH reactivation. Using this method via altering the reactivation temperature, microporous and mesoporous ACFs with similar SSA were produced [17].

In this work, highly microporous sawdust-based ACFs were prepared by KOH–steam reactivation. Our main objective was to investigate the pore formation mechanism during the reactivation process through the careful control of reactivation conditions. The pore structure and crystal structure of the prepared ACFs were characterized by nitrogen adsorption and Raman testing, respectively. Fourier transform infrared (FTIR) and X-ray photoelectron spectroscopy (XPS) were applied to characterize their surface chemical properties. The adsorption ability of the sawdust-based ACFs was evaluated by liquid adsorption of iodine.

## 2. Results and Discussion

### 2.1. Subsection Surface Morphology

**Figure 1:**
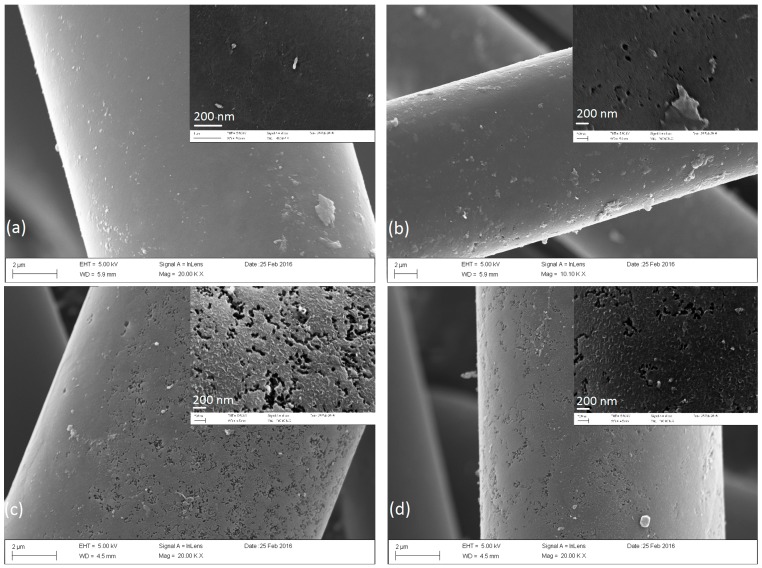
SEM photographs of surface of the activated carbon fibers (ACFs) before and after the steam reactivation. (**a**) A850-6-1; (**b**) A850-6-1-750-1; (**c**) A850-6-1-850-1; (**d**) A850-6-2-750-1.

Figure 1 shows the SEM micrographs of A850-6-1, A850-6-1-750-1, A850-6-1-850-1, and A850-6-2-750-1. Seen from the inset of Figure 1a, the surface of the fiber after KOH activation (A850-6-1) was rough, and there were obvious traces etched by KOH on the surface. When the activated fiber was reactivated by steam at 750 °C for 1 h, there were visible cavities and pores on its surface (A850-6-1-750-1, Figure 1b). When the activation temperature for steam reactivation was raised to 850 °C, large amounts of pores appeared on the fiber’s surface (A850-6-1-850-1, Figure 1c), revealing that activation temperature for steam reactivation had a significant effect on the development of porosity. Compared with A850-6-1-750-1, the sample A850-6-2-750-1 had a longer time for the first KOH activation. Correspondingly, A850-6-2-750-1 seemed to have a more rugged surface with cavities, indicating that the first KOH activation was the basis that determined the fiber porosity after the second steam activation.

### 2.2. Pore Structure

**Figure 2:**
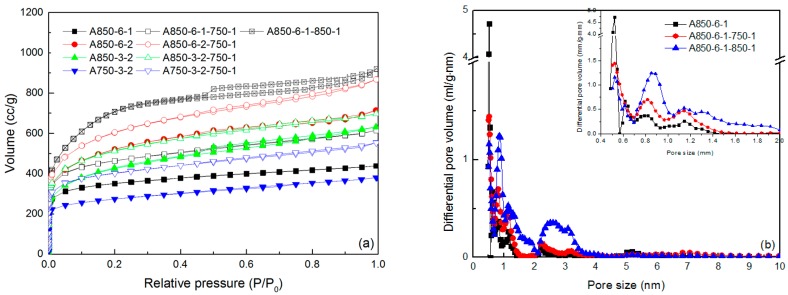

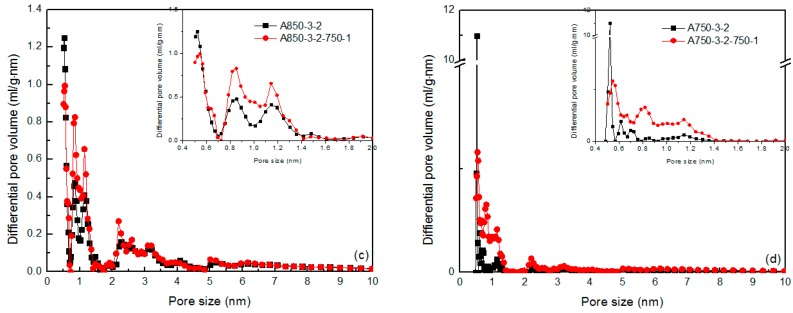
N_2_ adsorption/desorption isotherms (**a**) and pore size distribution (PSD) of the ACF sample A850-6-1 and its produced ACFs (**b**); A850-3-2 and its produced ACFs (**c**); and A750-3-2 and its produced ACFs (**d**).

Figure 2a presents nitrogen adsorption/desorption isotherms of KOH-activated ACFs and their derived ACFs with post-steam activation. The absorbed volume of nitrogen for all the samples after the second steam activation increased to some extent, indicating steam reactivation was available to further develop the porosity. Basically, the isotherms of KOH-activated ACFs belonged to typical Type I based on the IUPAC classification [18], where microporous adsorption is dominating. After steam reactivation, some of them exhibited a more open knee at the relative pressure range of 0–0.3, implying that large amounts of wider micropores were generated. The visible adsorption/desorption hysteresis loop could be observed for A850-6-1-850-1 and A850-6-2-750-1. All the isotherms of ACFs prepared by single steam activation in our previous study belonged to typical Type I and had no obvious hysteresis loop [8]. Therefore, compared with single steam or KOH activation, KOH–steam reactivation was able to add the mesoporosity to some extent.

The pore properties of the ACFs before and after the steam reactivation are listed in detail in Table 1. Both of the *S*_BET_ and the *V*_tot_ had a substantial increase for each sample after the steam reactivation. All of their *V*_meso_/*V*_tot_ values were below 50%, indicating that KOH–steam reactivation was an efficient method to develop the microporosity. Take the sample A850-6-1 for example. When it was reactivated by steam at 750 °C for 1 h, the *S*_BET_ and the *V*_tot_ increased from 1301 to 1715 m^2^·g^−1^ and from 0.679 to 0.952 cm^3^·g^−1^, respectively. Moreover, when it was reactivated at 850 °C, the *S*_BET_ was almost twice as high as that of the previous fiber. This value (2578 m^2^·g^−1^) was also much higher than that of ACFs (1882 m^2^·g^−1^) reported in our previous study, which were prepared by single steam activation at 850 °C for 1 h Additionally, the *V*_tot_ of A850-6-1-850-1 reached a high value of 1.425 cm^3^·g^−1^, higher than that of steam-activated ACFs (0.928 cm^3^·g^−1^). Although both of the *S*_meso_ and the *V*_meso_ increased to some extent, the *V*_meso_/*V*_tot_ decreased from 31.8% to 29.7%. According to this result, it can be speculated that the microporosity development had precedence over the mesoporosity development during the steam reactivation. According to our previous study, mesoporous ACFs prepared by steam–KOH reactivation (steam activation at 850 °C for 1 h and KOH reactivation at 850 °C for 1 h) exhibited a high *V*_tot_ of 1.389 cm^3^·g^−1^, where the *V*_meso_ contributed 64.1% [17]. This again confirmed that the mechanisms of pore fomation and development between steam–KOH reactivation and KOH–steam reactivation. Figure 2b shows the pore size distribution of A850-6-1 and its produced ACFs by steam reactivation using the quenched solid density functional theory (QSDFT) method. The micropores mainly concentrated at 0.5 nm for the KOH-activated ACF, while micropores at this region had a sharp decrease for the steam-reactivated ACFs no matter what temperature was selected. However, there were more micropores distributing at the range of 0.7–2.0 nm. In particular, the distribution peak around 0.9 nm for the sample A850-6-1-850-1 replaced that around 0.5 nm, becoming the main region where most micropores concentrated. As for the mesopores, there were apparently more mesopores distributing at the range of 2–4 nm. Based on these results, it can be concluded that most micropores generated near 0.5 nm during the first KOH activation. Further steam reactivation widened the micropore diameters and generated large amounts of micropores, resulting in the decrease in micropores at 0.5 nm. Meanwhile, the mesopores at 2–4 nm saw a slight increase. Especially steam reactivation at high temperature (850 °C) led to a remarkable increase in micropores. In contrary, potassium vapor derived from the reaction between KOH and C promoted the generation of mesopores during KOH reactivation at above 800 °C when the steam–KOH reactivation method was exploited. In other words, KOH–steam reactivation makes great contributions towards developing the microporosity, while steam–KOH reactivation mainly contributes to mesoporosity development. The change of PSD for samples AS850-3-2 and AS750-3-2 are presented in Figure 2c,d, respectively, which also confirmed the above conclusion. The yield of ACFs via reactivation had a great decrease even at a high reactivation temperature, which can be attributed to the fact that more carbon was eroded to develop the porosity (Table 1).

### 2.3. Chemical and Crystal Structure

**Figure 3:**
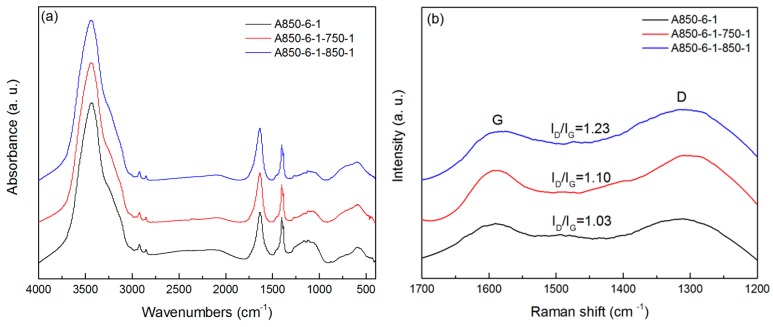
(**a**) Fourier transform infrared (FTIR) and (**b**) Raman spectra of A850-6-1 and its produced ACFs.

The 4000–400 cm^−1^ infrared spectral region of A850-6-1 and its produced ACFs via steam reactivation at 750 and 850 °C is shown in Figure 3a. For the KOH-activated ACF sample, the main peaks at 3435 and 1400 cm^−1^ were attributed to the stretching vibration and in-plane deformation of hydroxyl [19]. The peaks at 2922 and 2852 cm^−1^ were assigned to the symmetric and asymmetric stretching vibration of methylene [20]. Some weak peaks at 1300–1000 cm^−1^ can be identified as oxygen-containing functional groups such as C=O and C–O. After steam reactivation, no new peaks were generated, nor did old peaks disappear, indicating that steam reactivation did not alter the chemical structure of ACF. Moreover, the intensity of peaks at 1300–1000 cm^−1^ seemed to decrease gradually as the reactivation time increased, suggesting that more oxygen-containing functional groups were removed during steam reactivation, especially at high temperatures. This meant that the liquefied wood-based ACFs became more non-polar after reactivation. It was reported that, for a nonpolar, small molecule adsorbate such as iodine, non-polar activated carbon with high carbon content had a high adsorption capacity [21].

Figure 3b shows the Raman spectra of A850-6-1-750-1 and A850-6-1-850-1, as well as that of A850-6-1 as a reference, where two prominent peaks can be identified as the well-documented D and G vibration bands [22]. The ratios of the D/G band intensity (*I*_D_/*I*_G_) for A850-6-1 was near 1.0, while that for reactivated ACFs was above 1.1. Such an increase in *I*_D_/*I*_G_ indicated that defects at the edge of the pores produced by reactivation increased, which can be ascribed to the fact that more carbon atoms in the aromatic ring structure were attacked by the activating gas during the reactivation to produce micropores.

**Figure 4:**
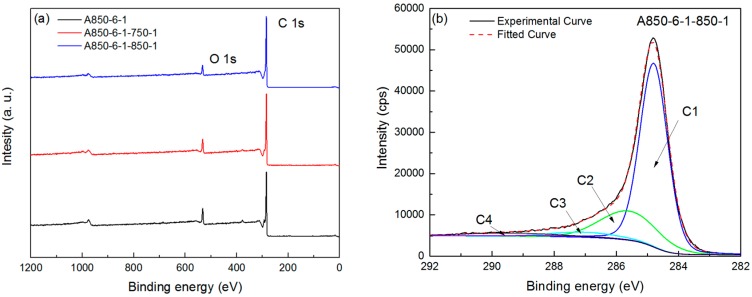
X-ray photoelectron spectroscopy (XPS) spectrum of A850-6-1 and its produced ACFs (**a**) and curve fitted high resolution XPS scans of A850-6-1-850-1 for C 1s (**b**).

Figure 4a presents the XPS spectra of A850-6-1-750 and A850-6-1, as well as that of A850-6-1 as a reference, demonstrating carbon and oxygen were the main elements of ACFs. Atomic concentrations on the surface of all the ACF samples are summarized in Table 2. All the KOH-activated ACFs showed an oxygen content decrease after the steam reactivation. As for A850-6-1, its oxygen content decreased from 15.0% to 12.7% after steam reactivation at 750 °C and then to 8.1% after steam reactivation at 850 °C. This indicated that more surface oxides were removed as temperature increased during steam reactivation, which was consistent with the FTIR analysis. The C 1s region of the XPS spectra of all samples is evaluated in Table 2, and the C 1s spectrum of A850-6-1-850-1 is presented in Figure 4b as an example. A decreasing trend was observed in graphitic carbon for all the KOH-activated ACFs after steam reactivation, whereas an opposite trend was observed in carbon bonded to oxygen-containing functions. Hence, Raman and XPS results further confirmed that the the reactions between steam and carbon resulted in the destruction of graphite-like microcrystallite structures and the formation of more micropores.

### 2.4. Iodine Adsorption

All the ACFs showed an increase in adsorption capacity for iodine after steam reactivation (Table 2). This variation of iodine adsorption of ACFs showed patterns similar to the *V*_micro_ values of N_2_ adsorption above. This can be attributed to the formation of micropores and the increase in surface area as a consequence of reactivation. For A850-6-1 with 1237 mg/g of iodine adsorption, the iodine adsorption of A850-6-1-750-1 and A850-6-1-850-1 reached 1628 and 1957 mg/g. The adsorption capacity for iodine achieved in this study was higher than that of biomass-based activated carbon from reedy glass (1128 mg/g) [23] and acorn shell (1209 mg/g) [24], as well as polymer-based ACFs from polyvinyl alcohol (PVA) (1934 mg/g) [2] and polyacrylonitrile (PAN) (1022 mg/g) [25].

## 3. Materials and Methods

### 3.1. Materials

The wood sawdust that originated from Chinese fir was obtained from a furniture factory (Beijing, China). Phenol (C_6_H_5_OH), phosphoric acid (H_3_PO_4_, 37 wt %), hexamethylenetetramine (C_6_H_12_N_4_), hydrochloric acid (HCl, 37 wt %), formaldehyde (CH_2_O, 37 wt %), potassium hydroxide (KOH), and iodine (I_2_) were purchased from Beijing Chemical Works. All the chemicals were all of reagent grade and were used without further purification.

### 3.2. Synthesis of Activated Carbon Fibers

The wood sawdust was firstly pulverized and screened to particle sizes of 60–80 meshes to prepare the precursor fibers through a series of processes including liquefaction, melt-spinning, and curing according to our previous study [8]. Prior to use, the wood sawdust was pulverized to powder and then screened to obtain particle sizes of 60–80 meshes. The mixture of wood flour, phenol, and phosphoric acid at a mass ratio of 1:5:0.4, loaded in a 500-mL three-neck glass flask, was heated in an oil bath at 160 °C for 2.5 h. Then, 5% hexamethylenetetramine as synthetic agent was added into the liquefied wood. The mixture was placed in a self-made spinning machine for heating from room temperature to 170 °C over 40 min. A melt-spinning apparatus was used to produce filaments. Afterwards, the resultant fibers were soaked in a solution with formaldehyde and hydrochloric acid (1:1 *v*/*v*) at 90 °C. After 2 h of this curing process, the fibers were rinsed several times with deionized water. An amount of 20 g of the resultant precursors were then carbonized in a horizontal transparent tube furnace (Y02PB, Thermcraft Inc., Winston Salem, NC, USA) at 500 °C in a N_2_ flow for 1 h of carbonization. A series of ACFs were prepared by a process of KOH activation with various activation temperatures (750 and 850 °C), KOH-fiber ratios (3 and 6) and activation times (1 and 2) from the carbon fibers. For a typical preparation, about 2 g of carbon fibers were put in the tube furnace after impregnation with KOH solution and were then heated to a specified activation temperature in a N_2_ flow at a heat rate of 4 °C·min^−1^. The obtained ACFs were washed with 1 M HCl and distilled water until a neutral pH was attained and dried at 103 ± 2 °C. The KOH-prepared ACFs were designated as A*T*_1_-*k*-*t*_1_, where *T*_1_ (750 and 850 °C), *k* (3 and 6), and *t*_1_ (1 and 2) represented the activation temperature, KOH/fiber ratio, and activation time for the KOH activation, respectively (Table 3). The KOH-activated ACFs were then put in a tube furnace, and the reactivation process was carried out at specified temperature (750 and 850 °C) in a steam–nitrogen gas mixture for 1 h. The steam-reactivated ACFs were denoted as A*T*_1_-*k*-*t*_1_-*T*_2_-*t*_2_, where *T*_2_ (750 and 850 °C), and *t*_2_ represented the activation temperature and time for the steam reactivation, respectively (Table 3).

### 3.3. Characterization of ACFs

Surface morphology of the ACFs was examined with a scanning electron microscopy (SEM) (Supra 40, Zeiss, Oberkochen, Germany). Nitrogen adsorption/desorption isotherm were collected at −196 °C on an Autosorb-iQ automatic analyzer (Quantachrome, Boynton Beach, FL, USA). The specific surface area (*S*_BET_) was calculated by the Brunauer–Emmett–Teller (BET) method based on nitrogen adsorption isotherm data [26]. The total pore volume (*V*_tot_) was evaluated by converting the amount of nitrogen adsorbed at a relative pressure of 0.995 to the volume of liquid adsorbate. The micropore area (*S*_micro_) and micropore volume (*V*_micro_) were obtained by the *t*-plot method [27]. The mesopore area (*S*_meso_) and mesopore volume (*V*_meso_) were calculated by Barrett–Joyner–Halenda (BJH) method. The PSD was obtained using the QSDFT method. The chemical groups of the ACFs were examined using FTIR spectrum analysis with a spectrometer (Tensor 27, Bruker Optics, Ettlingen, Germany) in the scanning range of 4000 to 400 cm^−1^. The samples were pulverized using size-100 mesh and mixed with potassium bromide at a ratio of 1:100 before being pressed into a disk. The Micro Raman spectrometer (RamanStation 400, PerkinElmer, Waltham, MA, USA) was used to investigate the conformation state graphite in the ACFs. XPS measurements were performed on an ESCALAB 250Xi spectrometer (Thermo Fisher Scientific, Waltham, MA, USA) using a monochromated Al Kα X-ray (*hv* = 1486.6 eV) source. A nonlinear least squares curve-fitting program (XPSPEAK software, Version 4.1) was used for XPS spectral deconvolution.

### 3.4. Liquid-Phase Adsorption of Iodine

Iodine number was determined according to the standard method specified by the American Society for Testing and Materials (ASTM) [28].

## 4. Conclusions

Bio-based ACFs with a highly microporous structure from sawdust were prepared with a simple reactivation method. The first KOH activation was responsible for generating most micropores of 0.5 nm and the second steam activation enlarged them and generated new large micropores of 0.7–2 nm. High reactivation temperature was able to develop the microporosity dramatically. The specific surface area and adsorption capacity for iodine of the ACFs can be greatly improved after reactivation. The produced sawdust-based ACF with cheap starting materials, highly microporous structure, and excellent iodine adsorption would be appropriate for industrial sorption processes.

## Figures and Tables

**Table 1:** Pore properties of the ACF samples.

Sample	*S*_BET_	*S*_micro_	*S*_meso_	*V*_tot_	*V*_micro_	*V*_meso_	*V*_meso_/*V*_tot_	*Yield*	*Iodine Adsorption*
	(m^2^·g^−1^)	(m^2^·g^−1^)	(m^2^·g^−1^)	(cm^3^·g^−1^)	(cm^3^·g^−1^)	(cm^3^·g^−1^)	(%)	(%)	(mg·g^−1^)
A850-6-1	1301	976	209	0.679	0.4	0.216	31.8	43.2	1237
A850-6-1-750-1	1715	1295	316	0.952	0.538	0.354	37.2	17.5	1628
A850-6-1-850-1	2578	2196	452	1.425	0.966	0.424	29.7	9.6	1957
A850-6-2	1861	1115	466	1.105	0.484	0.473	42.8	29.6	1713
A850-6-2-750-1	2153	1463	581	1.342	0.656	0.627	46.7	10.3	1895
A850-3-2	1512	909	572	0.977	0.402	0.549	56.2	32.4	1597
A850-3-2-750-1	1845	1241	586	1.08	0.542	0.519	48	16.7	1764
A750-3-2	1013	694	261	0.589	0.285	0.27	45.8	45.2	1044
A750-3-2-750-1	1493	1083	300	0.857	0.447	0.348	40.6	21.6	1566

**Table 2:** Results of the fits of the C 1s region for all the ACF samples.

Sample	Atomic Concentration (%)		Data Derived of C 1s Peaks (%)				
	C	O	C1	C2	C3	C4	C5
A850-6-1	85.0	15.0	67.1	16.6	6.1	4.3	5.9
A850-6-1-750-1	87.3	12.7	62.5	18.7	7.8	5.6	5.4
A850-6-1-850-1	91.9	8.1	59.6	20.1	10.8	9.5	-
A850-6-2	92.8	7.2	65.0	13.3	8.3	7.7	5.7
A850-6-2-750-1	93.5	6.5	61.4	17.6	11.8	-	9.2
A850-3-2	92.2	7.8	67.1	10.5	9.0	8.6	4.9
A850-3-2-750-1	93.1	6.9	63.2	15.6	9.2	7.5	4.5
A750-3-2	86.5	13.5	67.9	15.8	7.7	4.7	3.9
A750-3-2-750-1	88.4	11.6	65.3	14.7	8.5	5.6	5.9

**Table 3:** Conditions used for the KOH–steam reactivation.

Sample	KOH Activation			Steam Activation	
	KOH/Fiber Ratio	Temperature (°C)	Time (h)	Temperature (°C)	Time (h)
A850-6-1	6	850	1	-	-
A850-6-1-750-1	6	850	1	750	1
A850-6-1-850-1	6	850	1	850	1
A850-6-2	6	850	2	-	-
A850-6-2-750-1	6	850	2	750	1
A850-3-2	3	850	2	-	-
A850-3-2-750-1	3	850	2	750	1
A750-3-2	3	750	2	-	-
A750-3-2-750-1	3	750	2	750	1
